# Effectiveness of self-managed continuous monitoring for maintaining high-quality early essential newborn care compared to supervision visit in Lao PDR: a cluster randomised controlled trial

**DOI:** 10.1186/s12913-021-06481-6

**Published:** 2021-05-14

**Authors:** Sayaka Horiuchi, Sommana Rattana, Bounnack Saysanasongkham, Outhevanh Kounnavongsa, Shogo Kubota, Mariko Inoue, Kazue Yamaoka

**Affiliations:** 1 Center for Birth Cohort Studies, University of Yamanashi, 1110 Shimokato, Chuo-shi, Yamanashi Japan; 2 Department of Health Care and Rehabilitation, Ministry of Health, Ban thatkhao, Sisattanack District, Rue Simeuang, Vientiane, Lao PDR; 3 Department of Health Care and Rehabilitation, Ministry of Health, Ban Chomcheng, Sisattanack District, Rue Thadeua, Vientiane, Lao PDR; 4 Reproductive, maternal, newborn, child and adolescent health unit, World Health Organization Representative Office in Lao PDR, Saphanthongtai village, Saphanthong road, Vientiane, Lao PDR; 5 Teikyo University Graduate School of Public Health, 2-11-1 Kaga, Itabashi, Tokyo, Japan

**Keywords:** EENC, Neonatal care, Quality improvement, Resource-limited settings

## Abstract

**Background:**

Thousands of neonatal deaths are expected to be averted by introducing the Early Essential Newborn Care (EENC) in the Western Pacific Region. In Lao People’s Democratic Republic (Lao PDR), the government adopted the EENC programme and expanded it to district hospitals. With the expansion, maintaining the quality of EENC has become difficult for the government.

**Methods:**

A cluster randomised controlled trial with four strata based on province and history of EENC coaching was implemented to evaluate the effectiveness of self-managed continuous monitoring compared with supervisory visit in Lao PDR between 20 July 2017 and 2 April 2019. Health workers who were routinely involved in maternity care were recruited from 15 district hospitals in Huaphanh (HP) and Xiangkhouang (XK) provinces. The primary endpoint was the score on the determinants of EENC performance measured by the Theory of Planned Behaviour (TPB). Secondary endpoints were set as the knowledge and skill scores. A linear mixed-effects model was applied to test the effects of intervention over time on the endpoints.

**Results:**

Among 198 recruited health workers, 46 (23.2%) did not complete the final evaluation. TPB scores were 180.9 [Standard Deviation: SD 38.6] and 182.5 [SD 37.7] at baseline and 192.3 [SD 30.1] and 192.3 [SD 28.4] at the final evaluation in the intervention and control groups, respectively. There was no significant difference in changes between the groups in the adjusted model (2.4, *p* = 0.650). Interviews with participants revealed that district hospitals in HP regularly conducted peer reviews and feedback meetings, while few hospitals did in XK. Accordingly, in stratified analyses, the TPB score in the intervention group significantly increased in HP (15.5, *p* = 0.017) but largely declined in XK (− 17.7, *p* = 0.047) compared to the control group after adjusting for covariates. Skill scores declined sharper in the intervention group in XK (− 8.78, *p* = 0.026), particularly in the practice of managing nonbreathing babies.

**Conclusions:**

The study indicates that self-managed continuous monitoring is effective in improving behaviour among district health workers; however, additional measures are necessary to support its proper implementation. To maintain resuscitation skills, repeated practice is necessary.

**Trial registration:**

This trial was registered at UMIN Clinical Trials Registry on 15/6/2017. Registration number is UMIN000027794.

**Supplementary Information:**

The online version contains supplementary material available at 10.1186/s12913-021-06481-6.

## Background

Children are at the highest risk of death during the first month of their life. Approximately two and half million neonates, 7000 neonates per day, died in 2019 [1]. Neonatal deaths are mainly due to preventable causes such as premature birth, intrapartum-related complications, and neonatal sepsis [1].

Simple methods can prevent neonatal deaths in this critical period [2, 3]. The programme that has been widely spread in the Western Pacific Region to prevent neonatal deaths is the Early Essential Newborn Care (EENC) [4, 5]. More than 30,000 health workers in more than 3000 facilities have been trained under this programme within the region by 2017 [6].

Lao People’s Democratic Republic (Lao PDR), that had the highest neonatal mortality rate (28 neonatal deaths per 1000 live births) as of 2017 in the region [7, 8], tried expanding the EENC programme to the sub-national levels. With the expansion, monitoring and maintaining the quality of the EENC in each hospital, especially at the district level, became tremendously difficult for the government and central hospitals. Supervision strategies are commonly applied and considered the gold standard to promote performance and motivation of health workers; however, systematic reviews that examine the use of supervision as a method to improve the quality of care in low- and middle-income countries reported mixed evidence of the effect of supervision on quality improvement [9–11].

Lao PDR has only a limited capacity to implement routine supervisory visits, with supervisory visits from central and provincial hospitals to each district hospital occurring only once or twice a year based on their available budget. In addition, district hospitals act passively, making insufficient efforts to solve problems identified during supervisory visits, and eventually, the problems remain unresolved due to the lack of a follow-up mechanism to be implemented after supervisory visits [12]. It is desirable to establish a more sustainable and feasible mechanism to maintain the quality of neonatal care, especially in rural areas of resource-limited countries such as Lao PDR, to improve health outcomes of children.

We, therefore, examined the effectiveness of self-managed continuous monitoring involving periodical peer evaluation through direct observation and chart reviews as well as feedback meetings to analyse and plan actions to improve the quality of care compared with supervision to improve the quality of the EENC in Lao PDR.

## Methods

### Study design and settings

A cluster randomised controlled trial (cRCT) was implemented with the aim to evaluate the effectiveness of self-managed continuous monitoring (intervention) compared with supervisory visits (control). The self-managed continuous monitoring is an alternative monitoring method that is led by a district hospital. Based on the periodical peer evaluation through direct observation and chart reviews, quarterly feedback meetings are held to discuss measures for improvement. Contrarily, supervisory visit is implemented by provincial hospitals. Provincial supervisors provide advice for district health workers based on their evaluation during their quarterly visit. The study targeted 15 district hospitals in Huaphanh (HP) and Xiangkhouang (XK) provinces, located in the northern part of Lao PDR. The number of facility delivery in 2017 varied from 124 to 950 among the hospitals. It was registered at UMIN-CTR on 15/6/2017. Registration number is UMIN000027794. Details of the study methods are described elsewhere [13].

The cRCT was a two-armed parallel group study with four strata based on the province (HP / XK) and history of attendance in EENC coaching (Yes / No). Random numbers were generated within each stratum with a 1:1 allocation ratio using a permuted-block technique. Four district hospitals, two from each of the two provinces, received EENC coaching 12 months before the trial as pilot sites, whereas the remaining 11 district hospitals received EENC coaching for the first time before they were randomly allocated to the intervention and control groups. No refresh coaching for the four district hospitals was conducted.

During the trial period, self-managed continuous monitoring or quarterly supervisory visits was conducted in each district hospital allocated in the intervention and control groups, respectively. Health workers who were routinely involved in maternal and newborn care and received EENC coaching in the target district hospitals were eligible to join this study. Each district hospital has about 15 to 20 eligible health workers. Fifteen health workers were expected to be recruited in each district hospital to detect at least a 5-point increase in the score for determinants of EENC performance in the intervention group compared to the control group with 80% statistical power at a significance level of 0.05 using a two-tailed test. Study participants were monitored until 12 months after allocation or withdrawal for any reason from the study, whichever occurred earlier.

### Data collection

The determinants of EENC performance amongst health workers, the primary endpoint, were measured using the Theory of Planned Behaviour (TPB) [14, 15]. The TPB score was measured using a self-administered structured questionnaire designed to quantify determinants of behaviour: attitude, subjective norms, perceived behaviour controls, and behaviour intentions. A higher total score prospected a higher likelihood of staff practicing EENC in their daily work. The questionnaire was published elsewhere [13].

We set secondary endpoints as the changes in (1) EENC knowledge, (2) EENC skills, and (3) hospital environment facilitating implementation of EENC. At baseline and final evaluation, the knowledge and skills were quantified via written and simulation tests, and the hospital environment was reviewed by provincial facilitators using the standardised checklist. The written and simulation tests and checklist were published elsewhere [13]. In addition, interviews with health workers were performed at the final evaluation to review the degree of establishment of self-managed continuous monitoring (Additional file 1).

Participants and provincial facilitators were not blinded due to the nature of the intervention. Instead, team members who managed and analysed data were blinded until the code was open. Standardised checklists were used for outcome measurement to minimise bias.

### Statistical analyses

We examined the distribution and frequency of baseline characteristics of participants, summarised in percentage or mean [standard deviation: SD] and compared them between the intervention and control groups using the chi-square test or t-test to ensure successful cluster randomisation.

The primary endpoint, the change in the TPB score from baseline to 12 months after randomisation, was examined using an intention-to-treat (ITT) approach. A linear mixed-effects model employing the maximum likelihood method was applied by fitting the allocated group as a fixed effect and the district hospital as a random effect to test the effects of intervention over time. Crude and adjusted analyses were performed using the baseline TPB score, knowledge and skills, and other demographic variables. Missing data were imputed using the last observation carried forward method (ITT/LOCF). Sensitivity analyses were performed using the per-protocol set with complete data set (PPS/CDS). Changes in the TPB scores were further examined by dividing them into subdomains to identify factors that contributed to the improvement in the overall TPB score.

The same methods were applied to analyses of secondary outcomes, including changes in the scores of EENC knowledge and skills.

We further performed stratified analyses based on the province when there was strong evidence of the interaction between the effects of intervention and province.

A significance level of 5% with the two-tailed test was applied to all significance analyses. All statistical analyses were performed using SAS version 9.4 for Windows (SAS Institute, Cary, NC, USA).

## Results

**Fig. 1:**
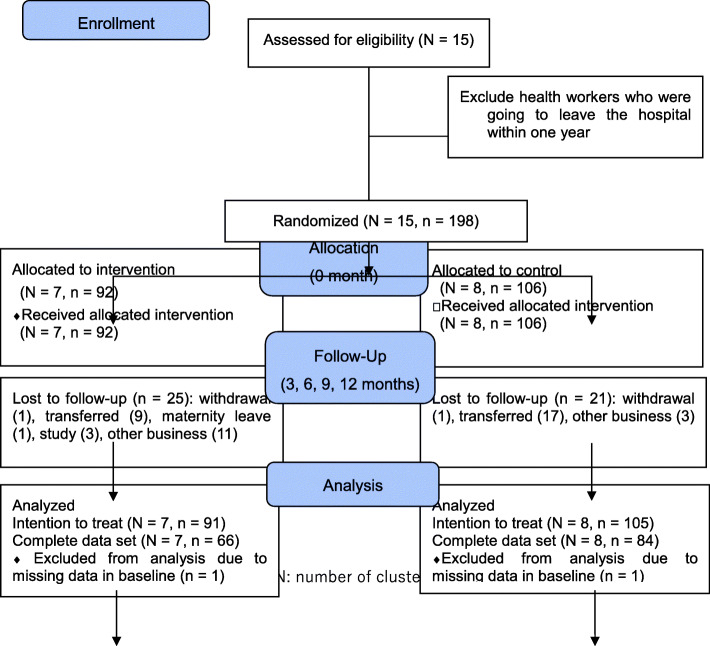
Study flow and participants

The study was started on 20 July 2017 and ended on 2 April 2019. A total of 198 health workers were recruited from 15 target district hospitals in the two provinces, of which 92 were allocated to the intervention group and the remaining 106 were allocated to the control group (Fig. 1). Two were excluded from the analysis due to missing data at baseline. In 196 health workers, 91 in the intervention group and 105 in the control group were included in the intention-to-treat analyses. Forty-six health workers out of 198 (23.2%) did not complete the final evaluation due to other commitments on the evaluation day, relocation, further training/study, maternity leave, or withdrawal, therefore they were excluded from the complete data set.

There was no significant difference in characteristics between the intervention and control groups, except for age and number of children (Table 1). Because a strong relationship between age and number of children was observed (Pearson correlation: r = 0.79, *p* < 0.001), we decided to omit the number of children from further multivariate analyses.

**Table 1:** Background characteristics of study participants allocated to the intervention and control groups

	Intervention group (***n*** = 91)	Control group (***n*** = 105)	***p***-value
**Age (year)**	31.5 [7.9]	34.4 [9.5]	0.021☨
**Sex**			
Female	65 (71.4%)	80 (76.2%)	0.449^§^
Male	26 (28.6%)	25 (23.8%)	
**Ethnicity**			
Lao	52 (57.1%)	68 (64.8%)	0.275^§^
Other minorities	39 (42.9%)	37 (35.2%)	
**Marital status**			
Married	71 (78.0%)	89 (84.8%)	0.224^§^
Single/divorced	20 (22.0%)	16 (15.2%)	
**Number of children**	1.1 [1.1]	1.7 [1.4]	0.002☨
**Position**			
Technical	72 (79.1%)	87 (82.9%)	0.505^§^
Management	19 (20.9%)	18(17.1%)	
**Title**			
Midwife	20 (22.0%)	24 (22.9%)	0.618^§^
Doctor	30 (33.0%)	27 (25.7%)	
Nurse	31 (34.0%)	44 (41.9%)	
Others	10 (11.0%)	10 (9.5%)	
**Experience (years)**	5.7 [6.5]	7.5 [8.3]	0.110☨

Data are mean [SD] or number (%)

*SD* Standard deviation

^§^ Chi-squared test was applied to examine the difference in two categorical data

☨t-test was applied to examine the mean difference between the two groups

### Change in determinants of EENC practice (TPB score)

The total TPB scores were 180.9 [SD 38.6] and 182.5 [SD 37.7] at 0 month and 192.3 [SD 30.1] and 192.3 [SD 28.4] at 12 months in the intervention and control groups, respectively, when the ITT/LOCF was applied (Table 2). Tests to determine the effect of intervention over time were not significant either in the crude analysis (Model 1) (1.7, *p* = 0.776) or in the model adjusted for the TPB score at baseline, gender, age, baseline skill and knowledge, ethnicity, position, job title, and years of experience (Model 4) (2.4, *p* = 0.650). When interaction between intervention and time as well as that between intervention and province were considered, the province strongly affected the effect of intervention on the TPB score (*p*-value = 0.005). Based on this result, we further conducted a stratified analysis by province.

**Table 2:** TPB scores at 0 and 12 months and their relation with the intervention group

	Intervention	Number	Mean [SD]		Model 1^a^		Model 2^a^		Model 3^a^		Model 4^a^	
			0 month (baseline)	12 months (endpoint)	Estimation	***P***-value	Estimation	***P***-value	Estimation	***P***-value	Estimation	***P***-value
**ITT (LOCF)**	**Self-monitoring**	91	180.9 [38.6]	192.3 [30.1]	1.7	0.776	1.7	0.738	2.4	0.650	2.4	0.650
	**Supervisory visit**	105	182.5 [37.7]	192.3 [28.4]								
**PPS (CDS)**	**Self-monitoring**	66	183.2 [39.6]	193.2 [29.1]	3.4	0.628	3.4	0.559	3.7	0.539	3.7	0.538
	**Supervisory visit**	84	186.3 [35.1]	192.9 [24.4]								

Model 1: crude mixed-effects model

Model 2: mixed-effects model adjusted for baseline TPB

Model 3: mixed-effects model adjusted for baseline TPB, gender, age, baseline skill and knowledge

Model 4: mixed-effects model adjusted for baseline, gender, age, baseline skill and knowledge, ethnicity, position, job title, and years of experience

*SD* Standard deviation, *ITT* Intention to treat, *LOCF* Last observation carried forward, *PPS* Per-protocol set, *CDS* Complete data set

^a^Test for the effect of intervention over time

In HP, TPB scores increased in both groups: from 179.3 [SD 42.0] to 202.8 [SD 26.0] in the intervention group and from 183.1 [SD 40.6] to 191.8 [SD 27.5] in the control group (Table 3). However, the intervention group saw a greater increase by 15 points over time in the TPB score compared to the control group (15.5, *p* = 0.017 in Model 4). Contrarily, in XK, the TPB score in the intervention group decreased from 183.2 [SD 33.4] to 177.2 [SD 29.5] in 12 months, whereas the score in the control group increased from 181.4 [SD 31.7] to 193.3 [SD 30.4]. There was a significantly larger reduction in the TPB score in the intervention group during 12 months compared to the control group in the adjusted mixed-effect analysis (− 17.7, *p* = 0.047 in Model 4). The results were similar in analyses with PPS/CDS (Additional file 1).

**Table 3:** TPB scores according to the intervention group by province (LOCF)

Province	Intervention	Number	Mean [SD]		Model 1^a^		Model 2^a^		Model 3^a^		Model 4^a^	
			0 month (baseline)	12 months (endpoint)	Estimation	***P***-value	Estimation	***P***-value	Estimation	***P***-value	Estimation	***P***-value
**Huaphanh**	**Self-monitoring**	54	179.3 [42.0]	202.8 [26.0]	14.8	0.054	14.8	0.020	15.5	0.017	15.5	0.017
	**Supervisory visit**	70	183.1 [40.6]	191.8 [27.5]								
**Xiangkhouang**	**Self-monitoring**	37	183.2 [33.4]	177.2 [29.5]	−17.9	0.068	−17.9	0.036	−17.7	0.047	−17.7	0.047
	**Supervisory visit**	35	181.4 [31.7]	193.3 [30.4]								

Model 1: crude mixed-effects model

Model 2: mixed-effects model adjusted for baseline TPB

Model 3: mixed-effects model adjusted for baseline TPB, gender, age, and baseline skill and knowledge

Model 4: mixed-effects model adjusted for baseline, gender, age, baseline skill and knowledge, ethnicity, position, job title, and years of experience

*LOCF* Last observation carried forward, *SD* Standard deviation

^a^Test for the effect of intervention over time

The trends of TPB scores in subdomains stratified by province are shown in Table 4. In HP, attitude significantly increased in the intervention group than in the control group by 1.53 points after adjusting for covariates (*p* = 0.002). In XK, there was a significant decrease in control belief in the intervention group compared to the control group (− 9.08, *p* = 0.025).

**Table 4:** TPB scores in each subdomain according to the intervention group by province (LOCF)

Province	TPB domains	Intervention	Number	Mean [SD]		Crude^a^		Adjust^ab^	
				0 (baseline)	12 months	Estimation	*P*-value	Estimation	*P*-value
**Huaphanh**	**Attitude**	Self-monitoring	54	25.8 [3.1]	27.1 [2.2]	1.53	0.008	1.53	0.002
		Supervisory visit	70	27.4 [1.7]	27.2 [1.9]				
	**Behavioral beliefs**	Self-monitoring	54	51.9 [14.0]	58.0 [9.6]	2.85	0.308	2.85	0.216
		Supervisory visit	70	53.4 [15.0]	56.7 [9.3]				
	**Subjective norms**	Self-monitoring	54	17.5 [3.4]	18.0 [2.9]	0.209	0.762	0.209	0.735
		Supervisory visit	70	18.0 [2.9]	17.6 [2.9]				
	**Normative beliefs**	Self-monitoring	54	44.2 [21.4]	53.8 [12.8]	5.08	0.188	5.08	0.120
		Supervisory visit	70	47.2 [17.8]	51.7 [15.6]				
	**Perceived behavioral control**	Self-monitoring	54	19.1 [3.6]	20.9 [2.6]	−0.43	0.592	−0.43	0.550
		Supervisory visit	70	19.3 [3.7]	21.5 [4.1]				
	**Control beliefs**	Self-monitoring	54	7.2 [15.0]	11.8 [14.7]	5.65	0.115	5.65	0.065
		Supervisory visit	70	4.5 [19.3]	3.4 [12.8]				
	**Intention**	Self-monitoring	54	13.5 [1.0]	13.8 [0.6]	−0.08	0.726	−0.09	0.666
		Supervisory visit	70	13.4 [1.7]	13.7 [0.9]				
**Xiangkhouang**	**Attitude**	Self-monitoring	37	26.8 [2.0]	26.2 [2.6]	−0.11	0.870	− 0.11	0.857
		Supervisory visit	35	27.5 [1.4]	27.1 [1.9]				
	**Behavioral beliefs**	Self-monitoring	37	52.7 [11.4]	52.7 [11.8]	−2.31	0.487	−2.31	0.436
		Supervisory visit	35	54.5 [11.0]	56.7 [10.2]				
	**Subjective norms**	Self-monitoring	37	17.6 [2.8]	16.9 [2.9]	−0.88	0.332	−0.88	0.273
		Supervisory visit	35	18.0 [2.9]	18.2 [2.8]				
	**Normative beliefs**	Self-monitoring	37	46.8 [17.1]	44.0 [15.3]	−5.10	0.343	−5.10	0.264
		Supervisory visit	35	47.4 [19.6]	49.7 [15.0]				
	**Perceived behavioral control**	Self-monitoring	37	19.9 [3.4]	21.1 [3.1]	−0.47	0.650	−0.47	0.593
		Supervisory visit	35	20.0 [4.1]	21.7 [2.8]				
	**Control beliefs**	Self-monitoring	37	6.1 [20.2]	3.0 [12.8]	−9.08	0.053	−9.08	0.025
		Supervisory visit	35	0.3 [19.1]	6.2 [16.6]				
	**Intention**	Self-monitoring	37	13.3 [1.2]	13.2 [1.4]	0.06	0.856	0.06	0.842
		Supervisory visit	35	13.7 [0.7]	13.6 [0.8]				

*LOCF* Last observation carried forward, *SD* Standard deviation

^a^Test for the effect of intervention over time

^b^Adjusted for baseline score

### Change in knowledge scores

Overall, there was no significant difference in the change in knowledge scores by intervention groups (0.31, *p* = 0.695 in Model 4) (Table 5). The same results were observed after stratifying by province (HP: 0.27, *p* = 0.796, XK: 0.43, *p* = 0.700 in Model 4). The results were similar in analyses with PPS/CDS (Additional file 1).

**Table 5:** Knowledge scores according to the intervention group (LOCF)

	Intervention	n	Mean [SD]		Model 1^b^		Model 2^b^		Model 3^b^		Model 4^b^	
			0 month (baseline)	12 months (endpoint)	Estimation	*P*-value	Estimation	*P*-value	Estimation	*P*-value	Estimation	*P*-value
**Overall**^a^	Self-monitoring	90	24.9 [4.7]	21.9 [5.1]	0.22	0.799	0.22	0.780	0.27	0.726	0.31	0.695
	Supervisory visit	104	25.0 [4.4]	21.8 [4.8]								
**Province**												
Huaphanh	Self-monitoring	54	26.4 [2.8]	23.6 [4.8]	0.30	0.796	0.30	0.775	0.22	0.830	0.27	0.796
	Supervisory visit	69	25.2 [4.5]	22.1 [5.1]								
Xiangkhouang	Self-monitoring	36	22.5 [6.0]	19.4 [4.6]	0.12	0.927	0.12	0.917	0.43	0.707	0.43	0.700
	Supervisory visit	35	24.5 [4.2]	21.3 [4.1]								

Model 1: crude mixed-effects model.

Model 2: mixed-effects model adjusted for baseline knowledge.

Model 3: mixed-effects model adjusted for baseline knowledge, TPB, and skill, gender, and age.

Model 4: mixed-effects model adjusted for baseline knowledge, TPB, and skill, gender, age, ethnicity, position, job title, and years of experience.

*LOCF* Last observation carried forward, *n* number, *SD* Standard deviation

^a^Test for the effect of intervention by province in the overall analysis produced a *p*-value of 0.027 after adjusting for baseline knowledge, skill, and TPB, gender, age, ethnicity, position, job title, and years of experience. ^b^Test for the effect of intervention over time

### Change in skill scores

In stratified analyses by province, both provinces showed decreasing trends in scores over time in both intervention groups (Table 6). Although there was no significant difference in the reduction between the two groups in HP, the decline was sharper in the intervention group than in the control group in XK (− 8.78, *p* = 0.026 in Model 4). In XK, there was a large reduction in the intervention group, particularly in managing nonbreathing babies. The results were similar in analyses with PPS/CDS (Additional file 1).

**Table 6:** Skill scores according to the intervention group stratified by province (LOCF)

Province	Skill	Intervention	n	Mean [SD]		Model 1^a^		Model 2^a^		Model 3^a^		Model 4^a^	
				0 month (baseline)	12 months (endpoint)	Estimation	*P*-value	Estimation	*P*-value	Estimation	*P*-value	Estimation	*P*-value
Huaphanh	Total	Self-monitoring	53	101.3 [6.3]	86.1 [16.3]	0.68	0.832	0.68	0.824	0.68	0.825	0.68	0.826
		Supervisory visit	69	98.7 [10.0]	82.8 [15.7]								
	Breathing baby	Self-monitoring	53	42.4 [2.8]	39.7 [4.7]	0.28	0.831	0.28	0.818	0.28	0.817	0.28	0.819
		Supervisory visit	69	41.9 [3.1]	38.5 [5.1]								
	Nonbreathing baby	Self-monitoring	53	58.9 [3.9]	46.4 [12.6]	−2.49	0.338	−2.49	0.310	−2.49	0.314	−2.49	0.317
		Supervisory visit	69	56.8 [7.4]	44.3 [11.8]								
Xiangkhouang	Total	Self-monitoring	36	90.3 [16.3]	75.2 [13.7]	−8.78	0.061	−8.78	0.023	−8.78	0.024	−8.78	0.026
		Supervisory visit	33	92.3 [22.1]	86.0 [9.9]								
	Breathing baby	Self-monitoring	36	39.3 [6.4]	34.5 [5.6]	−4.00	0.047	−4.00	0.014	−4.00	0.015	−4.00	0.017
		Supervisory visit	33	39.3 [7.7]	37.9 [4.1]								
	Nonbreathing baby	Self-monitoring	36	51.0 [11.1]	40.7 [9.1]	− 6.81	0.047	−6.81	0.011	− 6.81	0.011	−6.81	0.013
		Supervisory visit	33	53.0 [14.9]	48.1 [6.4]								

Model 1: crude mixed-effects model

Model 2: mixed-effects model adjusted for baseline skill

Model 3: mixed-effects model adjusted for baseline skill, TPB, and knowledge, gender, and age

Model 4: mixed-effects model adjusted for baseline skill, TPB, and knowledge, gender, age, ethnicity, position, job title, and years of experience

Test for the effect of intervention by province in the overall analysis produced a *p*-value of 0.007 after adjusting for baseline skill, TPB, and knowledge, gender, age, ethnicity, position, job title, and years of experience

*LOCF* Last observation carried forward, *n* Number, *SD* Standard deviation

^a^Test for the effect of intervention over time

### Change in a clinical environment over the study period

**Fig. 2:**
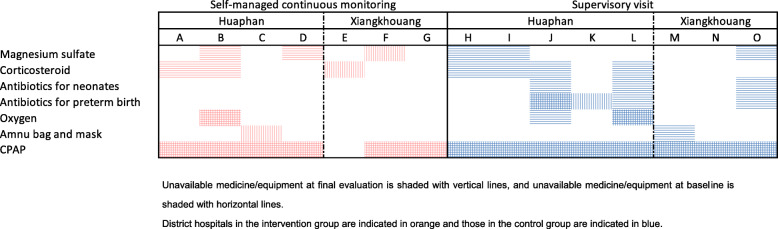
Availability of medicine/equipment at the baseline and final evaluation by intervention group and province

Between the baseline and final evaluation, improvement in the availability of essential medicine and equipment was observed in both groups in HP, but only in the supervision group in XK (Fig. 2). Among seven district hospitals allocated to the intervention group, two did not set up a committee for implementing self-managed continuous monitoring (Additional file 1). Two district hospitals set up a committee but not every relevant person was involved. The remaining three district hospitals set up a committee that included every eligible health worker. Two district hospitals implemented activities every quarter involving all the relevant colleagues. Overall, facilities in HP (A, B, C, and D) implemented monitoring activities better than the facilities in XK (E, F, and G).

## Discussion

The cRCT showed that there was no difference in the increase in TPB scores in 12 months in both self-managed continuous monitoring and supervisory visit groups. However, a significant interaction between the intervention and provinces was detected. The TPB scores increased more significantly in the intervention group than in the control group in HP, whereas they were lower in the intervention group than in the control group throughout the trial period in XK. The final evaluation suggested that the discrepancy between the two provinces is due to the existence of a strong EENC committee in a district hospital. District hospitals in HP conducted the monitoring activities by involving higher number of health workers within the hospital both in peer reviews and feedback meetings compared to district hospitals in XK, where only few workers were involved in the activities. We, therefore, consider that pooling effects of intervention among the two provinces will underestimate the real effect of intervention, and presenting results of stratified analyses by province is more appropriate.

Facilitation and follow-up from the provincial level have a significant influence on commitment and motivation of district health workers and development of a functioning EENC committee. Although the study protocol set up follow-up approach including frequency and timing, the implementation status differed between the two provinces. Provincial facilitators in HP set up a team within the provincial hospital and shared responsibilities to monitor the implementation of self-managed continuous monitoring in each district hospital allocated to the intervention group. By contrast, in XK, there was only one facilitator assigned to monitor the implementation of self-managed continuous monitoring with no backup for her work while she was absent for other commitments. As a result, there was no consistent follow-up for self-managed continuous monitoring from the province to district hospitals in the intervention group after the inception of the trial in XK, while supervisory visits were regularly implemented due to financial support. It suggests that it is also important to have a strong committee in the provincial level to monitor district hospitals. Future studies must consider standardisation and evaluate the facilitation and follow-up approach.

Among the subdomains of TPB scores, there was an increase in attitude and control beliefs in the intervention group in HP, whereas, in XK, control beliefs in the intervention group significantly decreased compared with the control group. Control beliefs may have increased among health workers in HP by solving identified problems by themselves. Improved availability of medicine/equipment may have also contributed to the increase. It suggests that support for ensuring an environment that enables routine provision of the EENC is important along with encouraging district health workers to perform EENC practice.

As for knowledge scores, there was no difference in the change of scores between the two groups in the overall analysis. After stratifying by province, the knowledge scores were higher in the intervention group in HP and lower in XK, but no statistically significant difference was observed. The scores changed almost consistently between the two groups in the overall and stratified analyses, indicating that the scores decreased by the same extent over time regardless of interventions in two provinces.

The skill scores decreased over time in both groups similar to the knowledge scores, but a larger decline was observed in the intervention group. The reduction was significant in practice for managing nonbreathing babies. The discrepancy in the change between the two intervention groups was more obvious in XK, with the scores in the control group being well maintained, while those in the intervention group declined. The larger reduction in the intervention group in XK may be due to poor implementation of the self-managed continuous monitoring. Practical skills, especially resuscitation skills, reportedly declined more rapidly than knowledge [16]. In particular, cases that needed resuscitation did not occur frequently because of the limited number of facility deliveries in the district level; therefore, none of the study participants practiced resuscitation during the trial period. This might have made it difficult to maintain resuscitation skills, especially for the intervention group, which could not receive direct advice from provincial facilitators [17]. Practice station with scenario cases will be helpful to maintain practical skills as a low-dose and high-frequency intervention along with positive feedback from trained facilitators [18–21]. The future studies need to incorporate these methods in the self-managed continuous monitoring.

The study indicated that self-managed continuous monitoring is effective to improve behaviour of health workers if it is properly implemented. However, we need additional measures such as practice station with scenario cases to improve practical skills, especially resuscitation skills. In the present study, the substantial differences in the implementation of self-managed continuous monitoring in the two provinces were observed due to difference in the commitment of the EENC committee in district hospitals. It was enhanced by the frequency of follow-up actions by provincial facilitators. District health workers need frequent contact with provincial facilitators to keep their motivation, which is consistent with studies that reported on the effectiveness of supportive encouragement on increased intrinsic motivation and leadership [22]. The results indicate that it is tremendously important to ensure understanding and commitments of provincial facilitators and standardise the follow-up approach before introducing the system to district hospitals.

The present study has several limitations. First, the study was implemented in two provinces in Lao PDR and the results revealed a significant difference in effects of the intervention between the two provinces. Therefore, the intervention might not be simply generalisable to other provinces. Consideration of the organizational culture of the province and adjustment of the coaching and follow-up actions would be necessary when introducing the intervention. Second, the low percentage of participants who completed the final evaluation is a shortcoming. Many health workers were transferred even though we limited our inclusion criterion to health workers without any plans of transfer at the baseline. As a result, we had fewer study participants than we expected, which might have reduced the power of the study. However, stratified analyses by province remain possibility of the positive effects of the self-managed continuous monitoring. For this point, further study is warranted. Third, the number of participants who dropped out of the study was higher in HP than in XK, which might have biased the results. However, analyses with CDS produced the similar results to that with LOCF, indicating that the drop-out did not cause substantial imbalance of participants between the two provinces. We believe that the results reflect the real situation in Lao PDR, which suffers from high turnover of health workers. Moreover, it was impossible to keep study participants blind regarding their allocated intervention due to characteristics of the intervention. Knowledge about their allocated intervention may have influenced health workers’ behaviour, however, we did not inform expected effects of the intervention to participants to minimise the potential bias. We think that the design is appropriate as a study to evaluate the effectiveness of the intervention in the real world. Finally, we could not evaluate the experience of postpartum mothers at hospitals, even though patient experience is considered an important dimension of quality care [23]. Further research that involves postpartum mothers will be needed.

## Conclusion

We conducted a cRCT with an objective to evaluate the effectiveness of self-managed continuous monitoring in improving the quality of EENC. The results indicate that the intervention is more effective in improving behaviour toward EENC practice among health workers compared to supervisory visit, provided the intervention is properly implemented. Development of a strong and motivated EENC committee within a district hospital, and standardisation of follow-up actions by provincial facilitators are keys for proper implementation. Frequent communications between the provincial facilitators and district health workers may help in development of a functioning EENC committee and improve implementation of the EENC.

## Supplementary Information

**Additional file 1 Annex1**. Interview questions. **Annex 2**. TPB scores according to the intervention group by province (CDS). **Annex 3**. Knowledge scores according to the intervention group (CDS). **Annex 4**. Skill scores according to the intervention group by province (CDS). **Annex 5**. Implementation status of self-managed continuous monitoring by district hospitals.

## Data Availability

The datasets used and/or analysed during the current study are available from the corresponding author on reasonable request.

## Abbreviations

CI: Confidence interval
cRCT: Cluster randamised controlled trial
EENC: Early Essential Newborn Care
HP: Huaphanh province
ITT: Intention-to-treat
Lao PDR: Lao People’s Democratic Republic
LOCF: Last observation carried forward
PPS/CDS: Per-protocol set with complete data set
SD: Standard deviation
TPB: Theory of Planned Behaviour
XK: Xiangkhouang province
