# Correction to: Effectiveness of self-managed continuous monitoring for maintaining high-quality early essential newborn care compared to supervision visit in Lao PDR: a cluster randomised controlled trial

**DOI:** 10.1186/s12913-021-06546-6

**Published:** 2021-06-28

**Authors:** Sayaka Horiuchi, Sommana Rattana, Bounnack Saysanasongkham, Outhevanh Kounnavongsa, Shogo Kubota, Mariko Inoue, Kazue Yamaoka

**Affiliations:** 1 Center for Birth Cohort Studies, University of Yamanashi, 1110 Shimokato, Chuo-shi, Yamanashi, Japan; 2 Department of Health Care and Rehabilitation, Ministry of Health, Ban thatkhao, Sisattanack District, Rue Simeuang, Vientiane, Lao PDR; 3 Department of Health Care and Rehabilitation, Ministry of Health, Ban Chomcheng, Sisattanack District, Rue Thadeua, Vientiane, Lao PDR; 4 Reproductive, maternal, newborn, child and adolescent health unit, World Health Organization Representative Office in Lao PDR, Saphanthongtai village, Saphanthong road, Vientiane, Lao PDR; 5 Teikyo University Graduate School of Public Health, 2-11-1 Kaga, Itabashi, Tokyo, Japan

**Correction to: BMC Health Serv Res 21, 460 (2021)**

**https://doi.org/10.1186/s12913-021-06481-6**

Following the publication of the original article [1], it was noted that Fig. 1 is disordered.

The correct Fig. 1 has been included in this correction, and the original article has been corrected.

**Fig. 1:**
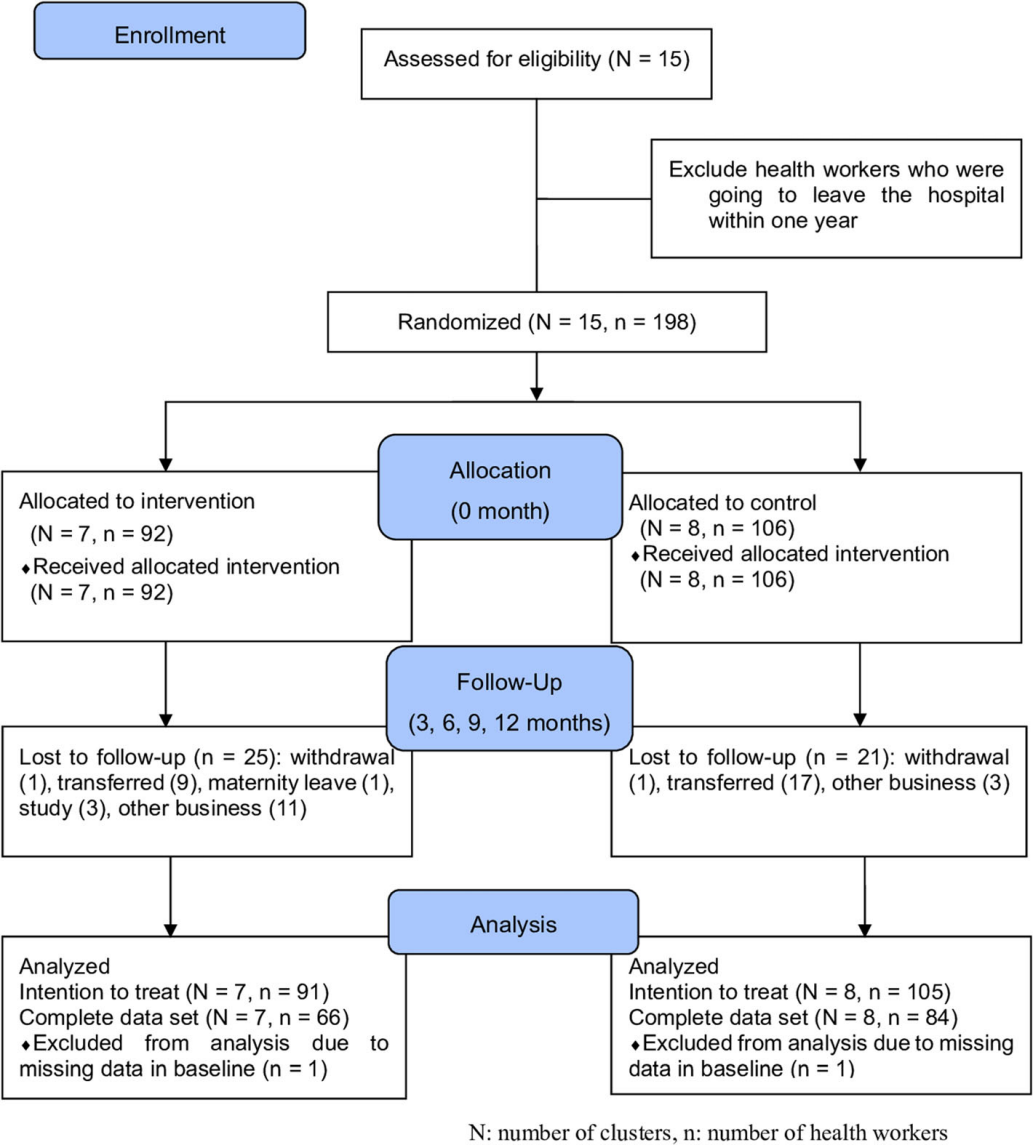
Study flow and participants
