# Supplementary material for: Effectiveness of self-managed continuous monitoring for maintaining high-quality early essential newborn care compared to supervision visit in Lao PDR: a cluster randomised controlled trial
Source: BMC Health Serv Res. 2021 May 14;21:460. doi: 10.1186/s12913-021-06481-6 (PMC8120813; doi:10.1186/s12913-021-06481-6)
Supplement: Supplementary file 1 — Additional file 1 Annex1. Interview questions. Annex 2. TPB scores according to the intervention group by province (CDS). Annex 3. Knowledge scores according to the intervention group (CDS). Annex 4. Skill scores according to the intervention group by province (CDS). Annex 5. Implementation status of self-managed continuous monitoring by district hospitals. [file 12913_2021_6481_MOESM1_ESM.docx]

Annex 2. TPB scores according to the intervention group by province (CDS)

| **Province** | **Intervention** | **Number** | **Mean [SD]** | | **Model 1**^☨^ | | **Model 2**^☨^ | | **Model 3**^☨^ | | **Model 4**^☨^ | |
| --- | --- | --- | --- | --- | --- | --- | --- | --- | --- | --- | --- | --- |
|  |  |  | 0 month  (baseline) | 12 months  (endpoint) | Estimation | p-value | Estimation | p-value | Estimation | p-value | Estimation | p-value |
| **Huaphanh** | **Self-monitoring** | 34 | 182.8 [44.5] | 208.4 [17.5] | 20.6 | 0.028 | 20.7 | 0.006 | 20.6 | 0.007 | 20.6 | 0.007 |
|  | **Supervisory visit** | 51 | 190.4 [37.0] | 195.4 [23.7] |  |  |  |  |  |  |  |  |
| **Xiangkhouang** | **Self-monitoring** | 32 | 183.6 [34.4] | 177.0 [30.4] | -15.6 | 0.138 | -15.6 | 0.081 | -15.2 | 0.105 | -15.2 | 0.106 |
|  | **Supervisory visit** | 33 | 180.0 [31.3] | 188.9 [25.4] |  |  |  |  |  |  |  |  |

CDS: Complete data set

SD: Standard deviation

☨Test for the effect of intervention over time.

Model 1: crude mixed-effects model

Model 2: mixed-effects model adjusted for baseline TPB.

Model 3: mixed-effects model adjusted for baseline TPB, gender, age, and baseline skill and knowledge.

Model 4: mixed-effects model adjusted for baseline, gender, age, baseline skill and knowledge, ethnicity, position, job title, and years of experience

Annex 3. Knowledge scores according to the intervention group (CDS)

|  | **Intervention** | **n** | **Mean [SD]** | | **Model 1**^☨^ | | **Model 2**^☨^ | | **Model 3**^☨^ | | **Model 4**^☨^ | |
| --- | --- | --- | --- | --- | --- | --- | --- | --- | --- | --- | --- | --- |
|  |  |  | 0 month  (baseline) | 12 months  (endpoint) | Estimation | p-value | Estimation | p-value | Estimation | p-value | Estimation | p-value |
| **Overall**^§^ | Self-monitoring | 67 | 24.5 [4.8] | 20.6 [4.6] | -0.03 | 0.972 | -0.03 | 0.968 | -0.04 | 0.968 | -0.04 | 0.968 |
|  | Supervisory visit | 84 | 24.7 [4.5] | 20.8 [4.6] |  |  |  |  |  |  |  |  |
| **Province** |  |  |  |  |  |  |  |  |  |  |  |  |
| Huaphanh | Self-monitoring | 36 | 26.3 [2.8] | 22.1 [4.8] | -0.01 | 0.993 | -0.01 | 0.993 | -0.01 | 0.993 | -0.01 | 0.993 |
|  | Supervisory visit | 51 | 24.9 [4.7] | 20.7 [4.9] |  |  |  |  |  |  |  |  |
| Xiangkhouang | Self-monitoring | 31 | 22.4 [5.7] | 18.8 [3.6] | -0.19 | 0.892 | -0.19 | 0.874 | -0.19 | 0.875 | -0.19 | 0.874 |
|  | Supervisory visit | 33 | 24.4 [4.3] | 21.0 [4.0] |  |  |  |  |  |  |  |  |

CDS: Complete data set, n: Number, SD: Standard deviation

^§^Test for the effect of intervention by province with the overall analysis produced a p-value of 0.077.

☨Test for the effect of intervention over time.

Model 1: crude mixed-effects model.

Model 2: mixed-effects model adjusted for baseline knowledge.

Model 3: mixed-effects model adjusted for baseline knowledge, TPB, and skill, gender, and age.

Model 4: mixed-effects model adjusted for baseline knowledge, TPB, and skill, gender, age, ethnicity, position, job title, and years of experience

Annex 4. Skill scores according to the intervention group by province (CDS)

| **Province** | **Skill** | **Intervention** | **n** | **Mean [SD]** | | **Model 1**^☨^ | | **Model 2**^☨^ | | **Model 3**^☨^ | | **Model 4**^☨^ | |
| --- | --- | --- | --- | --- | --- | --- | --- | --- | --- | --- | --- | --- | --- |
|  |  |  |  | 0 month  (baseline) | 12 months  (endpoint) | Estimation | p-value | Estimation | p-value | Estimation | p-value | Estimation | p-value |
| Huaphanh | Total | Self-monitoring | 34 | 101.2  [6.7] | 77.5  [13.8] | -2.21 | 0.541 | -2.21 | 0.514 | -2.21 | 0.517 | -2,21 | 0.520 |
|  |  | Supervisory visit | 51 | 98.1  [10.8] | 76.7  [12.9] |  |  |  |  |  |  |  |  |
|  | Breathing baby | Self-monitoring | 34 | 42.5  [2.8] | 38.2  [4.9] | 0.28 | 0.831 | 0.28 | 0.818 | 0.28 | 0.817 | 0.28 | 0.819 |
|  |  | Supervisory visit | 51 | 41.7  [3.4] | 37.2  [5.2] |  |  |  |  |  |  |  |  |
|  | Nonbreathing baby | Self-monitoring | 34 | 58.8  [4.4] | 39.3  [9.9] | -2.49 | 0.338 | -2.49 | 0.310 | -2.49 | 0.314 | -2.49 | 0.317 |
|  |  | Supervisory visit | 51 | 56.4  [7.9] | 39.5  [9.3] |  |  |  |  |  |  |  |  |
| Xiangkhouang | Total | Self-monitoring | 31 | 89.1  [16.9] | 71.6  [10.4] | -10.81 | 0.034 | -10.81 | 0.007 | -10.81 | 0.007 | -10.81 | 0.008 |
|  |  | Supervisory visit | 31 | 91.6  [22.7] | 84.9  [9.1] |  |  |  |  |  |  |  |  |
|  | Breathing baby | Self-monitoring | 31 | 38.8  [6.8] | 33.3  [5.0] | -4.00 | 0.047 | -4.00 | 0.014 | -4.00 | 0.015 | -4.00 | 0.017 |
|  |  | Supervisory visit | 31 | 39.1  [7.9] | 37.5  [3.9] |  |  |  |  |  |  |  |  |
|  | Nonbreathing baby | Self-monitoring | 31 | 50.2  [11.4] | 38.3  [6.6] | -6.80 | 0.047 | -6.80 | 0.011 | -6.81 | 0.011 | -6.81 | 0.013 |
|  |  | Supervisory visit | 31 | 52.5  [15.3] | 47.4  [5.8] |  |  |  |  |  |  |  |  |

CDS: Complete data set, n: Number, SD: Standard deviation

☨Test for the effect of intervention over time.

Model 1: crude mixed-effects model.

Model 2: mixed-effects model adjusted for baseline TPB.

Model 3: mixed-effects model adjusted for baseline TPB, gender, age, and baseline skill and knowledge.

Model 4: mixed-effects model adjusted for baseline, gender, age, baseline skill and knowledge, ethnicity, position, job title, and years of experience

Annex 5. Implementation status of self-managed continuous monitoring by district hospitals

| District hospital | Committee established | Percentage of health workers involved | Frequency of peer reviews | Frequency of feedback meetings (times/year) | Number of childbirths per year |
| --- | --- | --- | --- | --- | --- |
| A | Yes | 100 | Everyday | 4 and meetings with directors and all technical staff happened everyday | 270 |
| B | Yes | 77 | Everyday | 4 | 167 |
| C | Yes | 44 | 40 interviews and chart reviews, 37 direct observations | 4 | 180 |
| D | No (but assigned a responsible person) | 43 | 46 interviews and chart reviews, 26 direct observations | 4 | 397 |
| E | Yes | 36 | 20 interviews and chart reviews, 25 direct observations | 0 (only small meetings among involved staff) | 88 |
| F | Yes | 28 | 80 interviews and chart reviews, 40 direct observations | 4 and regular technical meetings happened every day | 281 |
| G | No | 13 | 5 cases per year | 0 | 399 |

Facilities A, B, C, and D are in Huaphanh province and the others are in Xiangkhouang province.
